# Supplementary material for: Are carbon emissions trading and green financial instruments synergistic? -Comprehensive quantitative research based on content analysis
Source: PLoS One. 2024 Mar 7;19(3):e0298601. doi: 10.1371/journal.pone.0298601 (PMC10919601; doi:10.1371/journal.pone.0298601)
Supplement: S1 Data — (ZIP) [file pone.0298601.s001.zip › Data/地级市Z_F工作报告.zip!/╔╜╢1⁄2/╢1⁄2╙¬╩╨/└┤╫╘╣1⁄2╓┌║┼í╛┬φ┐╦╩2╛▌═°í┐_2011.docx]

各位代表：

现在，我代表市人民政府向大会报告政府工作，请予审议，并请市政协委员和其他列席会议的同志提出意见。

一、“十一五”时期政府工作回顾

“十一五”时期，是东营发展历史上极不平凡的五年。五年来，在党中央国务院、省委省政府和市委的正确领导下，全市上下深入贯彻落实科学发展观，认真学习贯彻胡锦涛总书记视察东营时的重要讲话，解放思想，改革创新，攻坚克难，积极应对国际金融危机冲击，经济社会保持又好又快发展，黄河三角洲开发实现重大突破、上升为国家战略，“十一五”规划各项目标任务圆满完成。

经济总量快速膨胀，综合实力显著增强。初步统计，2010年全市生产总值达到2360亿元，比2005年翻一番，按可比口径，年均增长14.4%，城市综合竞争力列全国第25位。工业竞争力显著提升，全市规模以上工业实现主营业务收入6100亿元、增加值1740亿元，分别是2005年的3.8倍和2倍；收入过10亿元企业新增54家，过百亿元企业达到10家。经济效益大幅提高，全市实现工业利税1100亿元、利润720亿元，分别是2005年的1.9倍和1.6倍；地方财政收入104.88亿元，是2005年的2.7倍，年均增长22.2%。投资、消费不断扩大，“十一五”全社会固定资产投资4626亿元，是“十五”的2.5倍，年均增长21.6%；社会消费品零售总额378亿元，年均增长19.5%。金融机构各项存款余额1567.1亿元、贷款余额1148.1亿元，年均分别增长19.2%、22.3%。

经济结构不断优化，产业层次大幅提升。三次产业结构由4.2：79.7：16.1调整为3.6：72.7：23.7。石油工业稳步发展，地方经济快速突破，油地结构调整为3：7。市属经济快速发展，县域经济拉开新框架，经济结构明显优化。四大主体产业区实现重大突破。临港产业区，港口建设和园区配套取得重大进展，引进了一批大项目，被列为中国石油化工产业区。高端产业区实现跨越发展，五年生产总值、财政收入翻了两番，引进过亿元项目139个，电子信息、汽车及零部件、新能源等高端产业初具规模，东营经济开发区升级为国家级经济技术开发区。黄河口生态旅游区快速起步，游客综合服务中心、湿地公园建成投用，成为国家4A级景区。生态高效农业区成为新亮点，现代渔业示范区完成投资12亿元，14万亩养殖区投入生产，研发中心大楼投用；高效生态农业示范区、现代畜牧业示范区建设全面启动。工业结构调整成效显著，高新技术产业和先进制造业快速突破，大规模集成电路、广汽吉奥汽车、LED照明、光伏太阳能等项目相继落地，高新技术产业比重提高12个百分点；传统产业优化提升，化工、轮胎、纺织等产业80%以上的技术装备达到国内先进水平。服务业加速发展，文化旅游、现代物流、服务外包、金融保险等产业成为新的增长点，会展业快速起步，服务业比重提高7.6个百分点。科技创新能力不断提高，黄河三角洲可持续发展研究院等平台建设快速推进，组建了5个产业技术创新联盟，被评为国家知识产权工作示范城市。

“三农”工作不断加强，城乡统筹发展取得新成效。强农惠农政策全面落实，五年市财政支持“三农”资金35.77亿元，是“十五”时期的1.9倍。农业结构调整步伐加快，渔业、畜牧等优势产业快速发展，特色水产品养殖达到174万亩，标准化畜牧养殖小区达到1300个。农业产业化水平不断提高，农业龙头企业发展到508家，农民专业合作社发展到492个。品牌农业发展取得新成效，16个农产品获驰名商标和著名商标，黄河口大闸蟹入选中国十大名蟹。农业农村基础设施建设进一步加强，改造中低产田58万亩、灌渠10条，治理骨干河道334公里，维修病险水库7座，建设改造农村公路2889公里、危桥90座，新建农房5.3万户，改造危房1.6万户，新增沼气用户5.2万户，解决了93.8万农村人口的饮水安全问题。农村流通体系不断完善。农民科技培训力度加大，五年培训农民53.7万人次。县城和小城镇建设步伐加快，城中村改造和新型农村社区建设稳步推进，城乡一体化新格局初步拉开，全市城镇化率达到60%。

城市和基础设施建设取得重大突破，发展承载能力快速提高。广利河综合治理三年任务全面完成，“三园一区一岸线”工程取得重大成效，体育公园、文化公园和会展中心投入使用，科技公园软件大厦建成投用，清风湖景区成为全国首个新光源示范区，水城景观初步显现。黄河水城深度开发加快推进，金融商务中心、河海风情娱乐城、水城之窗、水城雪莲剧院等项目开工建设。五年新建改造城市道路280公里、桥梁38座，新增绿地1564万平方米，新建公园13座，市政公用设施不断完善，中心城建成区达到140平方公里。东营港2个3万吨码头建成投用，实现外轮开放，2个5万吨液体化工码头开工建设。广利港航道整治工程已经省发改委核准。东营机场4D级改造全面完成，新航站楼即将投用。威乌高速公路东营段建成通车，德大、黄大铁路开工建设。变电容量翻了两番，供电能力大大增强。“数字化东营”深入推进，信息化水平显著提高。

节能减排和环境保护力度加大，生态环境明显改善。节能降耗成效显著，实施节能技改项目362个，淘汰了一批落后产能。实施减排项目136个，新建改造污水处理厂8座、垃圾处理场5座，17家电厂全部完成脱硫改造，中心城区工业企业搬迁改造进展顺利，省控重点河流全部恢复鱼类生长。循环经济加快发展，建成了22个重点循环经济项目。全市万元生产总值能耗降低22%，二氧化硫和化学需氧量排放量分别下降38.65%和18%，圆满完成了“十一五”节能减排目标。“三网”绿化构筑起主体框架，累计植树7416万株，绿化面积72.5万亩，被命名为国家现代林业建设示范市。实施了刁口河流路恢复过水等工程，湿地生态环境进一步改善。

改革开放深入推进，体制机制创新取得重大进展。金融创新成效明显，引进了7家股份制银行，组建了3家村镇银行和1家农村商业银行，黄河三角洲产业投资基金获准筹建。财税体制改革力度加大，税收和非税收入征管机制进一步健全，财政管理制度不断完善。企业改革取得新成效，4家企业成功上市。农村综合改革继续深化，土地流转制度改革稳步推进，国有农场和集体林权制度改革进展顺利。医药卫生体制改革加快推进，基本药物制度试点成效明显，公立医院改革试点开始启动。文化体制改革扎实推进，组建了东营广播电视台、市演艺公司和文化市场综合执法机构。市县政府机构改革全面完成，事业单位分类改革扎实推进。对外开放不断扩大。全市进出口总值达到79.6亿美元，其中出口27.6亿美元，分别增长4.4倍和2.2倍。五年实际利用外资8.6亿美元，有6家世界500强企业落户东营。“走出去”步伐加快，境外投资、对外工程承包和劳务合作快速增长。

社会事业全面发展，和谐稳定局面进一步巩固。五年市财政投向社会事业60.76亿元。教育事业不断发展，学前教育、高中教育、职业教育得到加强，全部实现免费义务教育，被评为首批“全国推进义务教育均衡发展先进地区”。实施了校舍安全改造等工程，中心城区3座小学建成投用，技术学院和胜利一中迁建进展顺利。医疗卫生服务体系不断完善，建成了市人民医院病房楼、乡镇卫生院等卫生服务设施，实行了政府购买社区公共卫生服务。文化事业加快发展，建成了一批文化场馆，乡镇综合文化站实现全覆盖，成功举办了黄河文化旅游博览会等节庆活动，群众文化生活日益丰富；推出了《黄河入海流》等文化精品。全民健身活动蓬勃开展，竞技体育水平不断提高，成功举办了三届黄河口国际马拉松赛。人口和计划生育工作成效显著，连续保持低生育水平。新闻出版、文学艺术、社会科学等事业健康发展，国防教育、人民防空、民兵预备役、双拥共建和优抚安置工作成效明显，妇女儿童、青少年、残疾人、慈善、老龄事业持续发展，外事侨务、民族宗教、对台、档案、统计、史志、无线电管理、地名、仲裁、气象和红十字会等工作取得新成绩。精神文明建设得到加强，文明城市创建深入推进，“从我做起，走向文明”主题实践活动成效明显，市民文明素质有了新的提高。普法和依法治理深入开展，“五五”普法全面完成，基层民主不断扩大。建立了市长公开电话、市长市民对话会制度。平安东营建设全面推进，社会矛盾化解、社会管理创新、公正廉洁执法三项重点工作不断深化，信访工作得到加强，治安防控体系不断完善，基层单位内部安保工作扎实推进，严打整治斗争深入开展，社会秩序持续稳定。安全生产监管有力，事故起数和死亡人数连年下降。食品药品安全、重大疫病防控工作力度加大。应急管理体系得到加强。行政审批效率和服务水平进一步提高。民生持续改善，人民群众得到更多实惠。居民收入较快增长，城镇居民人均可支配收入23657元，农民人均纯收入8206元，年均分别增长9.6%和12.3%。就业再就业不断扩大，五年新增城镇就业20.1万人次，农村劳动力转移就业28.4万人次，城镇登记失业率控制在2%以内。社会保障体系更加完善，城镇居民医保参保率达到99.7%，新农合参保率达到99.6%，企业养老保险征缴率达到99.7%，工伤保险征缴率达到100%，新型农村养老保险试点扎实推进，失业保险覆盖面不断扩大；各类救助制度进一步健全，4次提高城市低保标准，3次提高农村低保标准和五保供养标准，5次提高失业保险金标准，连续5年上调企业退休人员待遇，两次提高物价补贴标准，去年底又对低收入群体发放了一次性物价补贴。保障性住房建设力度加大，建设保障性住房2.9万套、棚户区安置房3922套，支持油田建设职工住房3.2万套。城市社区集中整治全面展开，黄河南展区房台拓展和供水工程完工。圆满完成了援建北川任务，帮扶新疆疏勒和支援重庆酉阳工作扎实推进。

刚刚过去的2010年，是意义重大、很不平凡的一年。这一年，是“十一五”各项任务目标圆满收尾，东营的综合竞争力大幅提升的一年。这一年，是国家战略高点起步，开发建设顺利推进的一年。这一年，是结构调整成效明显，现代产业体系初具规模，全面拉开发展框架的一年。这一年，是财政收入突破百亿大关，质量效益不断提高，经济实力显著增强的一年。这一年，是城乡居民收入快速增长，民生保障不断加强，富民强市迈出更大步伐的一年。经过全市上下的共同努力，2010年的东营硕果累累、凯歌高奏，为“十一五”发展画上了圆满句号。

过去的五年，我们在科学发展观的指导下，坚持从东营实际出发，不断深化对市情的认识，着力提升发展境界，创新发展思路，谋划发展举措，着重把握了以下几个方面：

(一)抓住总战略不动摇，提升东营发展新境界。回顾“十一五”，我们最大的收获，就是把黄河三角洲开发建设确立为全市经济社会发展的总战略。2007年，在认真总结过去经验的基础上，经过充分研究论证，市委四届三次全体会议作出了加快推进黄河三角洲开发建设的决定，以此作为总战略，统领全市经济社会发展，举全市之力加快推进，以干促批。2009年11月23日，国务院批复了黄河三角洲高效生态经济区发展规划，黄河三角洲开发建设正式上升为国家战略。我们站在新起点，提升新境界，明确新目标，谋划新举措，着眼选准载体、重点突破，规划建设了四大主体产业区；着眼打造良好的开发环境，建设现代化黄河三角洲中心城市，实施了黄河水城、“三路两港一场”等重大工程；着眼建设生态文明，实施了以“三网”绿化为重点的生态建设工程，推动黄河三角洲开发进入了全新的历史时期。

(二)抓住创新进取不动摇，激发东营发展新活力。过去的五年，我们坚持在实践中探索，在创新中发展。连续三年开展了学习实践科学发展观活动，极大地解放了全市上下的思想，为科学发展和开发建设打开了思想通道。积极推进市场化运作，改革投融资体制，运用市场化手段成功实施了一批重大基础设施工程，组建了国有资产运营公司，形成了较强的融资能力。大力推进对外战略合作，支持企业强强联合，引进了一批国内外大企业集团。着力转变招商方式，突出产业招商和专业招商，整车制造、新能源装备、新信息等产业从无到有，快速起步。积极向上争取支持，向国家和省争取了一大批政策、项目和资金，为加快推进开发建设提供了有力支撑。

(三)抓住结构调整不动摇，增创东营发展新优势。无论外部环境如何变化，我们始终大力推进转方式调结构，在发展中转变，在转变中发展。2008年，金融危机席卷全球，面对突如其来的严峻挑战，我们沉着应对，迅速出击，全力打好扩大投资突破战、企业运行保卫战、结构调整攻坚战，充分利用金融危机的倒逼机制，加快转型发展，既有效化解了金融危机冲击，又提升了产业结构层次。我们不断深化对转方式调结构的认识，集中打好高端提升、重点带动、一体发展、绿色发展、创新驱动、增收富民六大转型攻坚战，狠抓节能、环保、安全生产三件大事，收到了明显成效。今天的东营，已经在资源型城市转型的进程中迈上新台阶，拉开了新格局，形成了新优势，为“十二五”发展奠定了坚实基础。

(四)抓住惠及民生不动摇，共享东营发展新成果。牢固树立执政为民理念，把保障改善民生作为一切工作的根本出发点和落脚点，着力解决人民群众最关心、最直接、最现实的利益问题。建立完善社会保障救助体系，扩大覆盖面，提高标准，东营各项民生保障指标均居全省前列，为困难群众和弱势群体撑起“保护伞”，让广大群众共享发展成果。

各位代表！过去的五年，是经济快速发展、竞争力大幅提升的五年，是城乡面貌发生深刻变化、人民生活水平显著提高的五年，是各项事业协调发展、社会全面进步的五年。我们在科学发展的道路上迈出了坚实步伐，落实胡锦涛总书记视察东营时的重要讲话结出了丰硕成果，应对国际金融危机冲击取得了重大胜利，推进黄河三角洲开发建设实现了重大突破。五年奋斗，五年辉煌。全市各级干部特别是基层干部牢记使命，创新进取，功不可没；广大企业特别是企业家勇担责任，迎难而上，贡献巨大；社会各界、人民群众维护大局，积极参与，无私奉献；油地军校、中央和省驻东营各单位通力协作，相互支持，共襄伟业；市人大及其常委会、人民政协和各民主党派围绕大局，议政监督，支持有力。在此，我代表市人民政府，向全市人民，向各民主党派、工商联、各人民团体和各界人士，向驻东营人民解放军、武警官兵和中央、省驻东营各单位，向所有为东营的建设和发展作出贡献的同志们、朋友们，致以崇高的敬意，表示衷心的感谢！

在充分肯定成绩的同时，我们也清醒地看到，我市经济社会发展还面临不少矛盾和问题。主要是，经济结构仍然不够合理，服务业和高新技术产业比重偏低，转方式调结构任务很重；高端人才不足，创新能力不强；生态环境脆弱，资源约束增强，节能减排压力巨大；社会事业发展滞后，城市功能不够完善；城乡差距仍然较大，部分群众生活还比较困难；一些领域的改革进展缓慢，体制机制活力不足；社会矛盾明显增多，维护和谐稳定的任务繁重；政府自身建设离人民群众的期望和要求还有很大差距，部分工作人员效率不高、执行力不强，素质能力有待进一步提高。对这些矛盾和问题，我们一定以高度负责的态度，采取更加有效的措施，努力加以解决。

二、“十二五”时期的奋斗目标和总体要求

展望“十二五”，东营正处于大有作为的黄金发展时期。黄河三角洲高效生态经济区、山东半岛蓝色经济区相继上升为国家战略，两大战略在东营融合交汇，为东营发展提供了千载难逢的重大机遇。经过“十一五”的努力，我市综合实力大大增强，基础设施更加完善，城市环境显著提升，内生动力明显增强，为“十二五”发展提供了坚实基础。当前，全市上下思想统一，油地军校团结一心，干部群众精神振奋，为“十二五”腾飞提供了良好的社会环境。我们一定要珍惜机遇、抓住机遇、用好机遇，坚定信心，乘势而上，更加奋发有为地推动经济社会又好又快发展。

“十二五”时期全市经济社会发展的指导思想是：高举中国特色社会主义伟大旗帜，全面贯彻党的十七届五中全会精神，以科学发展观为指导，深入贯彻落实胡锦涛总书记视察东营时的重要讲话精神，紧紧围绕科学发展这个主题，牢牢把握加快转变经济发展方式这条主线，按照“新起点新阶段新目标新局面”的工作要求，全力实施黄河三角洲开发建设总战略，着力调整优化经济结构，着力深化改革开放，着力加强生态建设，着力保障和改善民生，促进经济长期平稳较快发展和社会和谐稳定，为率先全面建成小康社会和国家级高效生态经济示范区打下坚实的基础。

综合考虑我市经济社会发展的趋势和条件，“十二五”时期的主要目标是：

——经济实力明显增强。全市生产总值年均增长12%，全社会固定资产投资年均增长18%，社会消费品零售总额年均增长17%，进出口总值年均增长10%，吸收外商直接投资年均增长10%，地方财政收入年均增长14%，经济发展质量和效益不断提高。力争经过五年的努力，经济总量翻一番，再造一个新东营。

——产业结构明显优化。传统产业加快改造提升，战略性新兴产业形成规模，服务业比重有较大提高，现代农业加快发展，三次产业比例调整为2.5：64：33.5。高新技术产业比重达到35%以上，形成高效生态产业体系。

——协调发展明显进步。地方经济占全市经济的比重逐年提高，资源型城市转型取得重大进展；区域经济协调发展，油地军校深度融合，新农村建设有较大突破，城镇化率达到65%，基本形成陆海统筹、城乡一体、区域协调发展的格局。

——生态环境明显改善。全面完成节能减排目标任务，城市污水、生活垃圾实现全收集、全处理，环境空气质量优良率达到85%以上，林木覆盖率达到25%，湿地生态功能显著增强，全面展现黄河水城、湿地之城、生态之城新形象。

——人民生活水平明显提高。城乡居民收入较快增长，城镇居民人均可支配收入年均增长11%，农民人均纯收入年均增长12%。就业持续增加，社会保障体系更加完善，社会事业加快发展，城乡公共服务体系基本健全，人民群众文明素质和健康水平进一步提高。社会管理体系不断完善，社会和谐进步，人民群众安居乐业。

各位代表，未来五年，将是东营大发展、大变化、大提升的五年，一个以高效生态为特色的经济新区将展现在世人面前，一个富裕文明和谐的经济文化强市将屹立在渤海之滨。到那时，经济综合实力大幅提升，高效生态发展模式基本形成，培育起一批过五百亿、上千亿的特大型企业集团，建成全省重要的先进装备制造业基地、精细化工基地、新能源基地和休闲度假胜地，成为全省新的经济增长极；到那时，城市综合服务功能大幅提升，立体交通网络发达，聚集辐射能力显著增强，成为区域性交通枢纽、金融中心、物流中心和科技创新中心，黄河三角洲中心城市的作用充分彰显；到那时，生态环境更加优美，人民生活更加富裕，社会更加文明和谐，成为独具魅力的适宜居住、适宜创业、和谐幸福的城市。

根据党的十七届五中全会精神和市委四届十一次全会通过的建议，市政府编制了《东营市国民经济和社会发展第十二个五年规划纲要(草案)》，已印发大会，请一并审议。

完成“十二五”时期的目标任务，必须坚持以科学发展为主题，推动经济社会各项事业沿着科学发展的轨道阔步前进。重点把握好四个方面：

——增强机遇意识、忧患意识、责任意识，全力实施国家战略。东营同时肩负着建设高效生态经济区和蓝色经济区两大战略任务。我们必须以强烈的责任感、紧迫感，雷厉风行抢抓机遇，求真务实用好机遇，高水平实施好国家战略。要坚持以科学规划引领开发，按照“黄蓝融合、陆海统筹、一体发展”的要求，两大战略统筹规划，整体推进。要坚持以主体产业区建设带动开发，重点突破“四区一城”，推动要素资源集聚，促进优势产业崛起；合理高效开发利用海洋资源，打造现代海洋产业聚集区。要坚持以完善的基础设施支撑开发，加快建设快捷畅通的交通体系、配套完善的水利体系、安全清洁的能源保障体系，提升开发建设的承载能力。要坚持以开放促开发，加大招商引资力度，加强对外战略合作，努力实现借力发展。

——突出高效生态，加快转变经济发展方式。加快转变经济发展方式是“十二五”发展的主线，我们要把转方式的要求贯穿于经济社会发展的全过程和各领域。打好结构调整攻坚战，按照传统产业高端化、新兴产业规模化、主导产业集群化的要求，着力发展环境友好型工业；加快突破现代服务业，促进服务业扩大总量、提升层次；以农业增效、农民增收为目标，加快转变农业发展方式，大力发展现代农业。牢固树立生态文明理念，构筑完善“五大生态系统”，推进经济社会生态化，创建国家生态市。积极推进新型城镇化，加快形成基本公共服务和基础设施一体化发展的新格局。提高自主创新能力，突破一批关键核心技术，推动经济发展向创新驱动转变。坚定不移实施人才强市战略，建设创新型人才队伍，形成人才辈出、人尽其才的良好局面。

——坚持以人为本，着力推进社会民生建设。始终把解决民生问题放在各项工作的首位，加快以改善民生为重点的社会建设，把更多财力投向民生领域。加强和改进公共服务，加快构建覆盖全体居民的终身教育体系、就业服务体系、社会保障体系、医疗保障体系、住房保障体系，努力满足人民群众的基本需求。全面提高社会管理水平，认真做好群众工作，统筹协调各方面利益关系，维护群众合法权益。扎实推进平安东营建设，完善社会治安防控体系，增强人民群众安全感。开展多种形式的和谐创建活动，努力形成社会和谐人人有责、和谐社会人人共享的生动局面。

——深化改革开放，不断增强发展动力和活力。要坚定不移深化改革，突出重点领域和关键环节，积极推进投资、金融、土地、财税、资源环境和行政管理等领域的改革，加快形成有利于科学发展和开发建设、充满活力的体制机制。坚持以开放促改革、促发展，把“引进来”和“走出去”更好地结合起来，全面提高开放型经济水平，形成参与国际经济合作和竞争的新优势。

各位代表！未来五年的蓝图已经绘就，美好前景令人鼓舞，神圣使命催人奋进。只要我们全市上下、油地军校、社会各界万众一心，众志成城，奋力开拓，我们就一定会开创科学发展和开发建设的新局面，“十二五”的奋斗目标就一定能够实现！

三、2011年政府工作任务

今年是“十二五”开局之年，也是实施国家战略的重要一年。做好今年的工作，意义十分重大。我们要抢抓机遇，积极作为，率先发展，为顺利完成“十二五”目标任务开好局、起好步，以优异成绩迎接建党90周年。

今年经济社会发展的预期目标是：全市生产总值增长12%，全社会固定资产投资增长19%，社会消费品零售总额增长17%，进出口总值增长12%，吸收外商直接投资增长10%，地方财政收入增长14%，城镇居民人均可支配收入增长11%，农民人均纯收入增长12%，城镇登记失业率控制在2.4%以内，居民消费价格总水平涨幅控制在4%左右。围绕实现上述目标，重点抓好九个方面的工作：

(一)全力实施国家战略，在高效生态经济区建设上实现新突破。新的一年，全力实施国家战略要重点抓好“四个突破”。全力突破四大主体产业区。临港产业区，坚持港区城一体发展，加快中海油、华泰、万达、海科等产业园建设，集中实施一批重大项目；加快完善基础设施，分步推进港区新城建设，增强承载发展能力。高端产业区，按照“新园区、新城区、新景区”的目标定位，突出抓好生态工业园、软件园、新能源产品及装备制造基地建设，实施好广汽吉奥汽车、电动汽车动力系统、LED背光模组、大规模集成电路等大项目，加快高端产业集群发展；规划建设黄河三角洲国际生态城。黄河口生态旅游区，突出湿地、鸟类、入海口三大特色，搞好游客综合服务中心、湿地公园管理运营，加强旅游产品开发和宣传推介，争创国家5A级景区。生态高效农业区，现代渔业示范区二期工程建成投用，完成商务中心、社区服务中心一期工程，启动蓝色小城镇和水产品大市场建设；加快高效生态农业示范区、现代畜牧业示范区规划建设，落地一批大项目。全力突破县域经济。各县区要立足实际，发挥优势，积极参与两大国家战略的实施，努力探索各具特色的发展模式；以省级经济开发区为重点，集中力量实施一批大项目好项目，培植主导产业，打造产业集群，壮大经济实力。全力突破蓝色经济。坚持开发与保护并重，围绕现代海洋渔业、海洋运输、滨海旅游等八大产业，积极推进大项目建设，培育新的经济增长点。全力突破开发机制。调整区域布局，优化组织结构，完善投入机制，以体制机制创新带动开发建设；深入研究和挖掘国家规划的政策潜力，积极争取更多的政策、项目和资金支持。油地军校团结结合是加快黄河三角洲开发建设的巨大优势。我们要高举团结发展旗帜，充分发挥胜利油田的产业、资金、技术优势，发挥石油大学的人才、科技优势，发挥济军生产基地的土地资源优势，推进资源整合、力量融合、工作结合，共促开发建设，共建美好家园。

(二)调整优化经济结构，在构建高效生态产业体系上实现新突破。调整优化经济结构，关键是立足我市产业基础，适应市场需求变化，瞄准高端产业发展前沿，加快构建高效生态产业体系。加快发展现代农业，带动农业结构调整。在稳定粮棉生产的同时，大力发展渔业、畜牧、林业、蔬菜、花卉和休闲观光农业六大产业。突出发展农业龙头企业，吸引国内外大企业参与设施农业开发，提高农产品转化加工能力，带动“农业三次产业”联动发展。继续抓好黄河口优势农产品品牌培育，搞好产销衔接，扩大市场份额，提高生产效益。加快发展农民专业合作社，重点培育50家市级示范社，带动农民参与产业化经营。加强农产品质量安全监管，规划建设黄河三角洲农产品综合检测中心，确保群众吃上放心食品。深入开展农民培训，建设中德黄河三角洲高效生态农牧业技术培训中心，提高农民实用技术水平。加强农业农村基础设施建设，突出抓好骨干排河及涝洼片治理、灌区及田间节水改造等水利工程，实施一批土地综合整治项目，提升农业机械化和信息化水平，提高农业综合生产能力。继续实施农村饮水安全工程，搞好农村电网续建配套，改善农村生活环境。认真落实各项强农惠农政策，大力发展农村二、三产业，增加农民收入。大力发展高新技术产业和先进制造业，带动工业结构优化升级。重点发展电子信息、新能源、新材料、新能源汽车等高新技术产业和战略性新兴产业，尽快培育成为支柱产业。加快发展汽车及零部件、石油装备、新能源装备等先进制造业，拉长产业链条，壮大产业集群。大力支持优势传统产业加快发展，继续实施“四个一”推进机制，用高新技术和先进适用技术改造提升化工、轮胎、纺织等产业，推动工业化与信息化深度融合，提升技术装备水平和产业层次，培育一批优势企业和产业。发挥企业在结构调整中的主体作用，尤其要发挥好大企业的表率和引导作用，以企业调整带动产业调整。

重点突破服务业，带动三次产业结构优化升级。把服务业发展摆上更加突出的位置，加强规划引导和政策支持，加大投入力度，加快壮大服务业规模，提高发展水平。着力抓好服务业城镇、园区、企业和项目建设，集中力量打造亮点。优先发展文化旅游业，集中突破黄河口生态旅游区、黄河水城旅游区、孙子文化旅游区三大景区，开发一批高水平旅游项目，举办一批重大文化旅游活动。大力发展现代物流业，重点建设海港、空港、中心城、广饶四大物流园区，引进和培育一批现代物流企业；加快黄河三角洲国际物流港和万通海欣保税罐区建设，争取开工建设保税物流中心。着力发展科技信息产业，依托东营软件园等平台，引进一批知名服务外包企业。促进金融、保险、房地产业健康发展。改造提升商贸餐饮等生活性服务业，加快发展商务服务、会展、休闲健身等新兴服务业。

(三)积极应对宏观环境变化，在促进经济平稳较快发展上实现新突破。保持经济平稳较快发展仍然是今年的重大任务，必须认真落实国家宏观调控政策，进一步加大投入、扩大消费、稳定外需，协调拉动经济增长。

加强牵动全局的大项目建设。大力开展“项目攻坚年”活动，将今年的经济社会发展任务实行项目化管理，靠项目的攻坚，带动经济社会又好又快发展。突出投资重点，优化投资结构，重点投向高端产业、社会民生、生态环保等领域。大力实施产业招商、专业招商，提高利用外资质量，重点加强与国内外大企业、大财团的战略合作，引进一批大、高、外项目。进一步完善项目推进机制，建立大项目直通车制度，促进各类项目加快建设。密切关注国家、省重点支持的项目，搞好项目论证，加大争取力度。优化投资环境，放宽市场准入，鼓励扩大民间投资。

扩大消费需求。认真落实国家扩大内需战略，把扩大消费与稳增长、控物价、惠民生结合起来。努力提高城乡居民收入，增强居民消费能力。扩大社会保障覆盖面，加强基本公共服务体系建设，改善群众消费预期。积极培植消费业态，扩大住房、汽车等大宗消费，重点开发旅游、文化等新的消费热点，推动消费升级。挖掘农村消费潜力，完善市场流通体系，继续推进农机下乡、家电下乡。进一步改善消费环境，保护消费者权益。认真落实稳定价格的政策措施，全面加强调控监管，保持物价总水平基本稳定。

提高外经贸水平。继续稳定和拓展外需，鼓励企业参加国际知名展会，建立境外营销网络，大力开拓国际市场。加快转变外贸增长方式，重点支持自主品牌和高附加值产品出口，促进加工贸易转型升级，增创外贸竞争新优势。帮助企业用好国家鼓励出口的各项政策，稳妥应对贸易摩擦。积极搭建对外经贸平台，继续办好国际石油石化装备展等大型展会。加大先进技术装备、关键零部件和紧缺物资进口，增加战略资源储备。加快“走出去”步伐，鼓励有条件的企业到境外承包工程、投资办厂，拓展发展空间。

强化财税金融保障。落实积极的财政政策，发挥财政政策在稳定增长、改善结构、调节分配、促进和谐等方面的作用。加大财源建设力度，依法加强税收和非税收入征管，保持财政收入稳定增长。优化财政支出结构，下决心压缩一般性支出，厉行节约，集中财力保重点、保民生。推进财税体制改革，规范财政分配关系，建立完善公共财政体系；适应国家税制改革的要求，推动二、三产业分离，培育壮大地方税源。认真落实稳健的货币政策，引导金融机构把握好信贷投放，加强对重点产业和薄弱环节的信贷支持，特别是加大对“三农”和中小企业的支持力度。完善政银企合作机制，加强信用担保体系建设，广开融资渠道。

(四)加强城市和重大基础设施建设，在提升承载能力上实现新突破。加快实施黄河水城深度开发，突出抓好清风湖景区、文化公园片区、科技公园片区、城市南展片区、滨海东城新区和广利河两岸片区等六大片区的综合开发，拉开城市新的发展格局。全面启动西城改造，搞好总体规划，分阶段确定改造重点，油地携手，市区联动，逐步推进。今年重点实施六大工程：一是公益场馆工程，加快建设水城雪莲剧院，开工建设奥体中心、儿童乐园、科技馆、图书馆、展览馆、博物馆、艺术馆。二是西城改造提升工程，重点抓好济南路提升改造、水污染综合治理等工程建设。三是城市道路工程，加快推进南三路和胜利大街、登州路、东一路、天目山路南延等道路建设。四是清风湖景区配套工程，开工建设清风阁、明月门、游客服务中心等项目，搞好周边环境提升改造。五是商务旅游设施工程，加快推进金融商务中心、河海风情娱乐城、揽翠湖景区建设。六是城市公用设施工程，实施南郊水厂增容等一批供水、供热、供电、供气设施项目。完成城市总体规划报批，编制中心城各类专项规划和南展区控制性详细规划。建立数字化城管系统，构建大城管格局，实现城市管理科学化、精细化。加强县城和小城镇规划建设管理，抓好城中村、城郊村、园区和大企业周边村改造，稳妥推进新型农村社区建设，加快城镇化进程。

加强重大基础设施建设。重点突破东营港，加快建设2个5万吨液体化工码头，开工建设2个5千吨液体化工码头，启动建设一批新的业主码头，规划建设防波堤和航道等基础设施；搞好已投用码头运营，扩大港口吞吐能力。完善广利港规划，引进战略合作伙伴，全面启动建设。加强东营机场配套完善，增开航线航班。积极配合德大、黄大铁路东营段施工，推进“小三线”地方铁路规划建设。开工建设东营至济南高速公路、飞机场到入海口旅游公路，积极推动沿海高等级公路规划建设。加快重大水利设施建设，启动南水北调配套工程，实施黄河清八河道治理，搞好东营港防潮堤、广利港临港新堤规划建设。加强能源建设，实施一批输变电工程。深入推进“数字化东营”建设，提高信息化水平。

(五)强化节能减排和环境保护，在生态环境建设上实现新突破。坚持生态立市、环境优先，加快生态化建设，确保完成省政府下达的节能减排任务。加大环境保护力度。严格执行环境影响评价和污染物总量控制制度，严禁新上高耗能高污染项目，全面整治污染企业。深入推进水气污染治理，重点治理广利河等流域和化工企业集中区，加强废气点源面源管理，控制城市扬尘、机动车尾气污染，建设改造10座污水处理厂、2座垃圾无害化处理场。加快中心城工业企业搬迁改造。加强农村面源污染治理和海域污染防治。加大节能降耗力度。积极推进土地、水、矿产、能源高效利用，突出抓好企业节能，加快推进建筑、交通和公共机构节能，推行合同能源管理，实施一批重点节能工程。大力发展循环经济，培育一批循环经济园区和生态工业示范园区，力争2012年建成国家级循环经济试点城市。加大生态建设力度。加快推进“三网”绿化工程，重点抓好干线路域、骨干水系、示范区和沿海防护林建设，新增绿化14.1万亩。积极创建国家级园林城市。加强湿地生态保护，重点抓好自然保护区湿地恢复，加快建设城东湿地公园，彰显城市湿地景观。积极推进社会生态化，抓好省级低碳生态示范城市创建工作，推广LED照明、地源热泵、光伏太阳能，普及应用节能新技术，引导全社会形成低碳生产生活方式和消费模式。

(六)实施科教兴市和人才强市战略，在创新型城市建设上实现新突破。提高科技创新能力。加大科技投入，完善落实鼓励科技创新的政策体系和激励机制。着力构筑创新平台，加快建设黄河三角洲可持续发展研究院、国家大学科技园及“生态谷”，规划建设科技教育园区，引进一批高层次研发及培训机构。强化企业在技术创新中的主体地位，支持骨干企业建设工程技术研究中心、企业技术中心。围绕优势主导产业发展，实施一批重大科技专项，新建一批产业技术创新联盟，提升产业核心竞争力。推进产学研紧密结合，加快科技成果转化。

推动教育改革发展。坚持教育优先发展，落实中长期教育改革发展纲要，提高义务教育水平，推动各级各类教育协调发展。大力发展学前教育，坚持政府主导，加强幼儿园规划建设管理，统一建园标准、收费标准和师资配置，坚决把这件关系子孙后代成长的大事办好。高度重视职业教育，支持东营职业学院搞好国家骨干高职院校建设，扶持市技师学院发展，建设黄河三角洲高级培训中心。支持高等教育和民办教育发展。完善学生资助制度，解决好困难家庭子女上学问题。加大教育投入，继续推进中小学校舍安全改造。加强教师队伍建设，提高教师综合素质。

建设人才强市。全面落实东营市中长期人才发展规划纲要，加大人才引进培养使用力度，统筹抓好企业经营管理人才、专业技术人才、高技能人才建设。着力引进急需高端人才，特别要引进一批产业领军人才，带动高端产业发展。推进政策和机制创新，加大知识产权保护和开发力度，创造人才发展的良好环境。

(七)积极推进文化建设，在发展先进文化上实现新突破。以建设文化强市为目标，促进文化事业和文化产业协调发展。突破文化设施建设，中心城集中抓好“三馆一院一中心”建设，农村集中抓好农村书屋、农村文化大院、广播电视村村通建设。实施一批重大文化产业项目，培育一批骨干文化企业，积极推进黄河口文化产业基地规划建设。加快推进国有经营性文化单位转企改制，全面推进文化市场综合执法改革。着力打造黄河口特色文化品牌。大力实施精品工程，积极推动文化创作。广泛开展群众文化活动和全民健身运动，加快竞技体育发展。繁荣发展哲学社会科学。加强社会主义核心价值体系建设，深入开展“从我做起，走向文明”主题实践活动，提高全社会文明程度。加强民主法制建设，扎实开展“六五”普法教育，扩大基层民主，做好村居换届选举工作。

(八)大力推进改革创新，在完善体制机制上实现新突破。着力推进金融创新，加快组建黄河三角洲银行，推动设立黄河三角洲产权交易市场，做好黄河三角洲产业投资基金募集发行工作，支持股份制银行健康运营，继续发展村镇银行、小额贷款公司等新型金融组织。深化投资体制改革，规范政府投融资平台运营，加强政府投资项目代建制管理。加快推进土地管理制度改革，组织开展好未利用地开发管理改革试点工作，稳步推进城乡建设用地增减挂钩试点。稳妥推进农村各项改革，坚持农村基本经营制度，积极探索农村土地流转和宅基地管理机制，加快推进农场改革、乡镇机构改革，搞好农村产权和集体林权制度改革。按照政事分开、政企分开、管办分离的原则，积极稳妥推进事业单位分类改革。建立资源节约与环境保护市场机制，完善资源有偿使用制度，探索建立生态补偿机制。

(九)保障改善民生，在和谐社会建设上实现新突破。积极扩大就业。重点做好高校毕业生、农村转移劳动力、城镇就业困难人员就业工作，年内新增城镇就业3万人，农村劳动力转移就业3.1万人，确保就业形势基本稳定。建立城乡、油地统一的人力资源市场，完善公共就业服务体系。落实自主创业税收优惠政策，引导和促进高校毕业生到基层就业和自主创业。加强就业再就业培训，大力开展就业指导和职业技能培训。完善就业援助制度，帮助零就业家庭和就业困难人员就业。建立完善企业与职工利益共享机制和劳动争议处理机制，维护劳动者合法权益。

健全完善社会保障体系。全面推行新型农村养老保险制度，年内在全省率先实现全覆盖。扩大城乡居民医疗保险覆盖面，继续做好医疗保险异地就医和企业养老保险转移接续工作。提高优抚对象生活补贴标准，做好城乡弱势群体、困难群众基本生活保障工作。提高企业最低工资标准。加强城乡保障性住房建设，加快实施棚户区改造工程。加快推进放心粮油食品工程和农户科学储粮工程。积极发展社会福利和慈善事业。

加快医疗卫生等事业发展。深化医药卫生体制改革。推进公立医院改革试点，鼓励社会资本举办医疗机构。全面推行基本药物制度。完善城乡医疗服务体系和公共卫生服务体系，规划建设市疾病预防控制中心。完善城乡药品供应保障体系。建立健全医疗救助基金，提高救助能力。全面做好人口和计划生育工作，继续稳定低生育水平，人口自然增长率控制在4.6‰以内。扎实开展双拥共建，做好国防教育、人民防空、民兵预备役等工作。关心支持妇女儿童、青少年、残疾人和老龄事业发展。积极推进外事侨务、民族宗教、无线电管理、档案、气象、对台、史志、仲裁、红十字会、防震和减灾救灾等工作。

加强和创新社会管理。健全基层管理和服务体系，强化社区管理职能，深入开展社区集中整治行动，提高社会管理水平。坚持和完善市长公开电话、市长市民对话会制度，畅通社情民意渠道。健全矛盾纠纷排查调处等制度，加强和改进信访工作，努力把各种矛盾纠纷化解在基层和萌芽状态。加强社会治安综合治理，依法打击各种违法犯罪活动，促进社会治安秩序持续稳定。加强突发事件应急管理，提高预防预警和处置能力。毫不放松地抓好安全生产，突出重点行业和领域专项整治，坚决防止重特大安全事故。强化食品药品安全监管，确保人民群众饮食用药安全。

四、切实加强政府自身建设

新的形势和任务，对政府工作提出了新要求。要切实加强自身建设，以更加开阔的视野、更加有力的举措、更加务实的作风，全面提高政府工作水平。

要增强群众观念。全体政府工作人员要牢固树立群众观念，时刻把群众的冷暖疾苦挂在心上，着力解决关系群众切身利益的问题。树立正确的政绩观，自觉站在群众的立场考虑问题，把群众满意作为工作标准。深入实际，深入基层，深入群众，真诚倾听群众呼声，真情体察群众疾苦，真实掌握群众愿望，更好地为群众服务。要尊重群众的主体地位，虚心听取群众意见，广泛采纳群众建议，组织开展好“两评一树”活动，做到问政于民、问需于民、问计于民。

要提高素质能力。全体政府工作人员要更加主动地加强学习，更加自觉地加强实践锻炼，着力提高谋划科学发展、推进开发建设的能力。要加强战略思维，善于研判形势，把握大局，增强工作主动性、预见性。要坚定改革创新、攻坚克难的信心决心，不断解放思想，更新观念，着力提高解决实际问题的能力，创造性开展工作。要高点定位，瞄准一流，创先争优，勇当排头，把东营的事业奋力推向前进。

要转变工作作风。全体政府工作人员要切实增强事业心和责任感，有敢于负责的境界和勇气，有敢于拼搏的激情和豪气，有敢于奉献的品格和正气，始终保持奋发有为、干事创业的精神状态。要大力弘扬求真务实作风，把心思放到干事业上，把功夫下到抓落实上，把本领用在促发展上，努力提高水平干出成效。坚持重实绩、办实事、求实效，多做打基础、谋全局、利长远的工作。政府各部门要树立全市“一盘棋”思想，自觉维护大局，加强配合，协调联动，形成工作合力。要提高工作效率，加强机关效能建设，推动政府工作提速。要提高执行力，完善目标责任制，加强督查考核，确保各项任务落到实处。

要坚持依法行政。认真落实依法行政实施纲要，严格按照法定权限和程序履行职责、行使权力。深入推进科学民主决策，建立完善重大决策听证和风险评估制度，提高行政决策的科学化、民主化、法治化。进一步规范行政执法，做到规范执法、公正执法、文明执法。深化行政审批制度改革，提高审批服务效率。加大政府信息公开力度，加强电子政务建设，提高权力运行的透明度。深入推进办事公开，提供便民服务。严格执行人大及其常委会的决议决定，支持人民政协履行职能，认真办理人大代表建议和政协提案，主动听取各民主党派、工商联、各人民团体和人民群众的意见建议，健全行政监督体系和问责制度，自觉接受各方面监督。

要加强反腐倡廉建设。坚持廉洁从政、从严治政，加强政风行风建设，健全完善惩治和预防腐败体系，进一步拓展从源头上防治腐败工作领域。深入开展反腐倡廉教育，筑牢拒腐防变的思想道德防线。加强制度建设，坚持用制度管权、管事、管人，加强对权力运行的监督和制约。继续加大案件查处力度，依法严惩腐败分子。全体政府工作人员特别是领导干部要严格执行党风廉政建设责任制规定和廉政准则，树立为民、务实、清廉的良好形象。

各位代表！东营的发展已经进入新的历史阶段，使命崇高，责任重大。让我们紧密团结在以胡锦涛同志为总书记的党中央周围，高举中国特色社会主义伟大旗帜，深入贯彻落实科学发展观，在省委、省政府和市委的坚强领导下，解放思想，抢抓机遇，团结奋斗，创新进取，合力谱写黄河三角洲开发建设的崭新篇章，共同开创东营繁荣富强、辉煌灿烂的明天！
